# Subjective and Objective Cancer‐Related Cognitive Impairments Among Systemic and Radiation Therapy‐Naïve Female Cancer Patients

**DOI:** 10.1002/cam4.70908

**Published:** 2025-04-22

**Authors:** Maria‐Chidi C. Onyedibe, Martina E. Schmidt, Pauline Bizer, Philipp Zimmer, Karen Steindorf

**Affiliations:** ^1^ Division of Physical Activity, Prevention and Cancer, German Cancer Research Center (DKFZ) and National Center for Tumor Diseases (NCT) A Partnership Between DKFZ and University Medical Center Heidelberg Germany; ^2^ Department Performance and Health TU Dortmund University Dortmund Germany

**Keywords:** anxiety, breast cancer, cancer‐related cognitive impairment, neuropsychological tests, sleep problem

## Abstract

**Background:**

Cancer‐related cognitive impairment (CRCI) is a frequent and burdensome problem that is still insufficiently understood and managed. We investigated subjective and objective measures of CRCI, as recommended by the International Cancer and Cognition Task Force (ICCTF) in cancer patients prior to systemic or radiation therapy with respect to potential influencing or associated psychosocial, demographic, or lifestyle factors.

**Methods:**

Female patients with breast or gynecological tumors (*n* = 239, mean age = 55.5, SD = 11.6) prior to any systemic or radiation therapy completed validated subjective (FACT‐Cog: perceived cognitive impairment [PCI], perceived cognitive ability [PCA], impact on quality of life [IQoL]) and objective measures of CRCI (Trail Making Test [TMT‐A and ‐B], Controlled Oral Word Association Test [COWA], and Hopkins Verbal Learning Test‐Revised [HVLT‐R]). Association with cross‐sectionally assessed age, body mass index, education, smoking, alcohol intake, sleep problems, social support, anxiety, and pain was investigated using multiple linear regression models.

**Results:**

A quarter (25.1%) of patients showed indication for CRCI based on the PCI score. Subjective and objective CRCI measures showed no or only weak correlations, also when adjusting for age and education (partial Spearman correlations with each other, all |*r*| ≤ 0.21). Anxiety, sleep problems, and pain were significantly associated with low subjective cognitive function (PCI, PCA, and IQoL). Poor objective cognitive values (TMT, COWA, and HVLT‐R) were mainly determined by higher age and lower education.

**Conclusions:**

Cancer‐related cognitive impairment is not solely (chemo‐)therapy‐induced but may be triggered or influenced by anxiety, sleep problems, and pain. Addressing these issues early in the treatment phase could potentially alleviate perceived CRCI. The ICCTF‐recommended neuropsychological tests do not adequately capture this CRCI prior to systemic or radiation therapy, but could serve as complementary tools to monitor cognitive changes over time, independent of psychosocial influences.

## 1 Introduction

Patients treated for cancer frequently report having trouble in remembering, thinking, concentrating, or finding the right words and this can persist even years after cancer therapy [1, 2]. Previously termed “chemobrain” and thought to be the result of chemotherapy treatments, research has shown that these symptoms could occur also with other therapies and even before cancer treatment [3]. Against this background, the International Cancer and Cognition Task Force (ICCTF) has agreed to refer to the symptoms as “cancer‐related cognitive impairments (CRCI)” and defined it as self‐reported cognitive complaints or a decline in memory, attention, concentration, and executive function among patients with non‐central nervous system cancers [4]. CRCI has been shown to impact patients' quality of life, disrupt their return to work, and lead to a decrease in self‐confidence at work or in social relationships [5, 6]. Due to the increasing number of cancer survivors, CRCI is becoming an emerging individual and public health burden [7].

In previous studies, the majority being conducted among breast cancer survivors, CRCI prevalence rates varied between 15% and > 50% [1]. Reasons for this huge variation may include different treatment settings, different assessment methods and cut‐offs, but also the lack of a clear definition of CRCI. CRCI is often assessed through patient‐reported outcomes, reflecting subjective perceptions, or objectively measured using standardized neuropsychological tests such as the Trail Making Test (TMT) [4]. The relationship between subjective and objective CRCI, however, is not fully understood. A systematic review exploring the relationship between subjective and objective CRCI reported that only 8 of 24 included studies found a significant association between subjective and objective measures of cognitive performance [8]. Subjective cognitive impairment experienced by patients appears often more severe than that objectively measured by neuropsychological test [9]. Different reasons for this heterogeneity are discussed. CRCI might comprise a large spectrum of problems such as, for example, impaired short‐term memory, reduced concentration, slower processing speed, or problems recalling names or words. The different measures of CRCI might, thus, represent different types or aspects of CRCI. The ICCTF recommends assessing subjective cognitive function with the FACT‐Cog questionnaire as well as objective functions using the TMT [TMT‐A and ‐B], Controlled Oral Word Association Test [COWA], and Hopkins Verbal Learning Test‐Revised [HVLT‐R], i.e., testing learning, memory, processing speed, and executive functions [10]. However, the meaning and informative value of the different measurements, and which ones are best for clinical use, are still not fully understood [11].

Likewise, the causes of CRCI are not well understood, but may be related to different factors, including the cancer itself, the effects of anesthesia or elevated stress due to cancer surgery, effects of cancer treatments, inflammation, hormonal changes, disturbed sleep, and the emotional and psychological stress associated with a cancer diagnosis [1, 3, 12]. Various psychosocial factors, such as social support, (trait) anxiety, as well as sleep problems and pain, and comorbid conditions such as depression and fatigue have been linked with CRCI, but the evidence is inconclusive [1]. Genetic predisposition might also play a role, as carrying specific genes, e.g., BDNF, COMT, or ApoE polymorphism, may increase the risk for CRCI [9, 13]. As recently concluded by an expert meeting of the National Cancer Institute, the broad term “CRCI” is likely an umbrella for several phenotypes and, thus, the most appropriate cognitive measure may depend on the suspected mechanism or type of interest and further patient characteristics [2].

Most studies were conducted to assess CRCI during or after cancer treatment. However, some studies showed that breast cancer patients already had more objective cognitive impairment before any cancer treatment compared to healthy controls [12, 14], or clinically meaningful impairments in self‐reported cognitive function measures [15]. In order to unravel the complex and likely multifaceted mechanisms underlying CRCI, it is advantageous to measure cognitive function prior to the initiation of any systemic or radiation therapy. This offers a valuable opportunity to investigate what else, besides chemotherapy and other systemic cancer treatments or radiation therapy, might contribute to cognitive problems. In particular, it allows for an examination of the ICCTF recommended measurements with regard to potential forms of CRCI that are not caused by therapy toxicities.

Hence, in this study, we investigated subjectively perceived and objectively measured CRCI as recommended by ICCTF in women with breast or gynecological cancer prior to undergoing any systemic or radiation therapy and potential influencing or predictive psychosocial, demographic, or lifestyle factors.

## 2 Materials and Methods

### 2.1 Participants and Procedures

The CogniFiT study recruited 239 women with breast or gynecological tumors at the National Center for Tumor Diseases (NCT) Heidelberg between November 2018 and March 2023. Participants were included if they were 18 years or above and were about to start therapy from the following categories: chemotherapy, radiotherapy, hormone therapy, or immunotherapy. Participants were excluded from the study if they had a previous cancer therapy within the last 2 years, had any known neurodegenerative diseases (e.g., Alzheimer's, Huntington's), psychiatric disorders, neurological damage (e.g., epilepsy, stroke), or were under medication shown to affect cognitive performance. We screened the medical records for eligible patients at the NCT Heidelberg, informed them personally or by phone call about the study, and asked them for participation.

### 2.2 Measures

Although CogniFiT is a longitudinal study, for the present research questions, we considered the baseline assessment prior to the start of any systemic or radiotherapy only. According to the recommendations of the ICCTF [10], we assessed the following subjective and objective measures of cognitive function:

#### 2.2.1 Functional Assessment of Cancer Therapy‐Cognitive Scale (FACT‐Cog)

The FACT‐Cog version 3 was used to assess self‐reported cognitive function [16]. FACT‐Cog is a validated 37‐item questionnaire created specifically to assess cognitive impairment in cancer patients. It consists of four subscales: perceived cognitive impairment (PCI—20 items), perceived cognitive ability (PCA—9 items), comments from others (4 items), and impact of cognitive impairment on quality of life (IQoL—4 items). All scores were linearly transformed to a 0%–100% scale, with higher scores indicating better cognitive function (i.e., lower subjective CRCI). We did not analyze the comments‐from‐others subscale due to highly skewed data, with 75% of participants reporting the maximum value, and because this subscale relies on reports from others. The developers of the FACT‐Cog questionnaire recommend reporting the PCI subscale as the main questionnaire outcome [17]. Van Dyk et al. [18] validated a cut‐off of 60 (raw score, corresponds with 75 on the 0–100 scale) for this subscale to discriminate CRCI cases from non‐cases. The FACT‐Cog has been validated several times with cancer patients [16], and demonstrated high internal consistency also in our data (Cronbach's alpha = 0.94, 0.94, and 0.92 for PCI, PCA, and IQoL, respectively).

#### 2.2.2 TMT

The TMT is an objective paper‐pencil test including two parts [19]. In TMT‐A, the participant must draw a line to connect consecutive numbers from 1 to 25, which are scattered across the sheet. In TMT‐B, the participant connects numbers and letters in an alternating progressive sequence, 1, A, 2, B, 3, C, etc. The times taken to complete each part are used to measure attention, psychomotor speed, and mental flexibility, with longer times indicating worse performance. The TMT‐B is more demanding, requires more mental flexibility, and is therefore more sensitive to executive functioning. The TMT shows adequate test–retest reliability and validity [20]. Age‐ and education‐matched normative values of the TMT‐A and ‐B [21] were used to identify indications of CRCI.

#### 2.2.3 The COWA

The COWA assesses verbal fluency, which is one of the most frequently used measures of executive function [22, 23]. A systematic review suggested that verbal fluency measures are sensitive, but not specific, indicators of frontal lobe damage [23]. The COWA has two components: a semantic fluency test and a phonemic fluency test. A German version of COWA (Regensburg fluency test) uses one letter from the three‐letter set containing “S”, “P”, and “M” in order to assess phonemic fluency under time constraints [22]. Specifically, individuals are given 120 s to name as many words as possible beginning with the given letter. In the semantic fluency test, persons are given 60 s to generate as many words as possible from a given category, selected from a three‐category set: Forenames, Animals, and Foods. Admissible responses are summed; higher scores indicate better cognitive function. The intercorrelations obtained for individual tasks based on a sample of 528 adults were between 0.60 and 0.72 for letters and between 0.48 and 0.72 for categories [22].

#### 2.2.4 HVLT‐R

The HVLT‐R is a brief word‐list learning and memory test with six alternate forms [24]. It consists of three free recall learning trials (asking to recall 12 words read aloud), a delayed recall trial after 20–25 min, and a recognition task (asking to identify which words from a subsequently presented list were among the previously shown 12 words). The HVLT‐R yields a number of raw and calculated scores including total recall score (sum of learning trials, scale 0–36 words), delayed recall score (0–12 words), percentage retention (0%–100%), and a recognition discrimination index (RDI, 0–12). The HVLT‐R has good test–retest reliability and demonstrated construct validity [24, 25]. Higher scores indicate better cognitive function.

#### 2.2.5 Assessment of Other Factors

The 19‐item Pittsburgh sleep quality index (PSQI) was used to assess sleep quality and disturbances during the past week [26]. The PSQI assesses subjective sleep quality, sleep latency (i.e., time spent falling asleep), sleep duration (i.e., total number of hours sleeping), sleep efficiency (i.e., hours of sleep divided by hours spent in bed), sleep disturbance (i.e., reasons sleep was interrupted), use of sleep aid medication, and daytime dysfunction over the previous 7 days. These seven components are each scored from 0 (no difficulty) to 3 (severe difficulty) and the sum of them yields the total score, with higher scores indicating more sleep problems. As with the other patient‐reported outcomes, the sum score was linearly transformed to a 0–100 scale. The PSQI has shown good psychometric properties in terms of its reliability and validity among breast cancer patients [27].

Anxiety was assessed by using the trait‐score of the State–Trait Anxiety Inventory (STAI‐T). The STAI‐T consists of 20 items on a four‐point Likert scale with higher scores indicating more anxiety [28]. The STAI‐T evaluates relatively stable aspects of “anxiety proneness”, encompassing general states of calmness, confidence, and security. This instrument has been widely used in cancer patients including breast cancer survivors [29].

Pain was derived from the according symptom item of the European Organization for Research and Treatment of Cancer Quality of Life Questionnaire (EORTC QLQ‐C30). According to the scoring manual, the pain score was calculated as the average of two items assessed on a four‐point Likert scale and transformed into a 0–100 score.

Fatigue was assessed using the EORTC QLQ‐FA12. The total fatigue score on a 0–100 scale was calculated according to the scoring manual, with higher values indicating greater levels of fatigue [30].

Depressive symptoms were measured with the revised 20‐item Center for Epidemiologic Studies Depression Scale (CESD‐R) [31]. Scores were transformed to a 0–100 scale, with higher scores indicating higher symptom burden.

The validated German version of the five‐item ENRICHD social support instrument (ESSI) was used to measure the participants' range of social support in their life [32]. The items of the ESSI are assessed on a five‐point Likert scale numbered 1 (None of the time) to 5 (All of the time). The individual scores are summed to create a total score, which is then transformed to a 0–100 scale, with higher scores indicating greater social support.

Information collected from the medical record included age, weight, and height (to calculate the body mass index, BMI). School and professional degree, current alcohol consumption, and current tobacco smoking were self‐reported by the participants via questionnaire. Education was categorized based on the highest school or professional degree as: academic (university degree), high (high school degree, referring to the German “(Fach‐)Abitur” or a degree of a specialized school, i.e., entitling access to universities), moderate (middle school or business school degree), or basic (any lower education than other categories).

### 2.3 Data Analysis

Descriptive analyses of the subjective and objective measures, and the proportion of patients with an indication for CRCI, e.g., scores below cut‐offs provided in the literature, were performed to enable interpretation of the CRCI measures [10, 18, 21]. Given the correlation of age and education with objective measures, partial Spearman correlation coefficients were calculated controlling for both age and education to present the correlation between subjective and objective CRCI measures.

Multiple linear regression was employed to examine the factors influencing both subjective and objective CRCI. The multiple regression analysis was conducted for each of the three subdimensions of subjective CRCI measures: PCI, PCA, and IQoL, as well as for the objective CRCI measures (TMT‐A, TMT‐B, and HVLT‐R: sums of learning trial, percentage retention, RDI, delayed recall, and COWA: semantic fluency test and a phonemic fluency). As independent factors of interest, we a priori considered social demographic factors (age and education), lifestyle characteristics (smoking, alcohol consumption, and BMI), and psychological predictors (social support, sleep problems, anxiety, and pain), which we simultaneously included in the models. As depression and fatigue were highly correlated with subjective cognitive outcomes (Spearman correlations of −0.62 to −0.72, Table S1) and may share common aetiologies with CRCI or affect each other mutually, they might blur (joint) underlying factors when included in the model. Thus, models were calculated both without and with depression and fatigue. Collinearities and normality of residual assumption were checked, and there were no deviations. All analyses were conducted using SAS Version 9.4.

## 3 Results

### 3.1 Description of the Study Population

Table 1 outlines the study participants' characteristics. The majority of participants had invasive breast cancer; only three women had a ductal carcinoma in situ, and three had a gynecological tumor. Only 3 patients had metastatic disease. Patients were on average 55.5 years of age (standard deviation, SD = 11.6) with an age range of 24–84. Most patients (95.4%) had surgery, with a mean of 42 days before baseline, while 1 patient had no surgery due to metastatic disease and 10 patients were scheduled to receive neoadjuvant therapy.

**TABLE 1:** Characteristics of the study population (*n* = 239).

Characteristics			
Age	Mean (SD)	55.5	(11.6)
BMI	Mean (SD)	25.8	(5.5)
Tumor entity	Breast, invasive	229	95.8%
	Breast, in situ	3	1.3%
	Gynecological	7	2.9%
Metastases	No	236	98.7%
	Yes	3	1.3%
Cancer surgery	No	9	3.8%
	Yes	230	96.2%
*If yes*: days since surgery	Mean (SD)	41.3	(17.4)
Education^a^	Basic	25	10.5%
	Moderate	78	32.6%
	High	51	21.3%
	Academic	85	35.6%
Smoking	Never	114	47.9%
	Former	90	37.8%
	Current	34	14.3%
Children < 18 years	No	174	72.8%
	Yes	65	27.2%
Married/with partner	No	54	22.8%
	Yes	183	77.2%
	Missing	2	
Sleep problems	Mean (SD)	33.0	(18.0)
Anxiety	Mean (SD)	32.2	(19.1)
Pain	Mean (SD)	31.1	(30.2)

*Note:* Sleep (PSQI, total score), pain (EORTC QLQ‐C30 symptom score), and anxiety (STAI‐T) are transformed to a 0–100 scale.

^a^ Education was categorized based on the highest school or professional degree as follows: academic (university degree), high (high school degree, referring to the German “(Fach‐)Abitur” or a degree of a specialized school, i.e., entitling access to universities), moderate (middle school or business school degree), or basic (any lower education than other categories).

### 3.2 Descriptive Analyses of the Subjective and Objective Cognitive Measures

The distributions of the subjective and objective cognitive measures are presented in Table 2. Based on the PCI cut‐off reported in the literature [18], 25.1% of patients could be considered as having (subjective) cognitive impairment. According to the threshold of clinical importance for the cognitive function assessed by the EORTC QLQ‐C30 [33], 38.4% had clinically relevant poor cognitive function in our study sample before the start of cancer therapy. Regarding objective cognitive measures, the proportion of patients with indications of a potential cognitive impairment was lower based on single TMT‐A (8.9%), TMT‐B (10.6%), and HVLT‐R sum (0.8%) or delayed recall (1.7%) measures using the criterion of values beyond 2 SD of the normative mean. Considering TMT‐A and ‐B together, with the ICCTF criterion [10] that both tests were beyond 1.5 SD or at least one of both tests beyond 2 SD of the normative mean, resulted in 17.5% of patients with an (objective) indication of cognitive impairment. However, very little overlap was noted between these subjective and objective CRCI cases, i.e., only 5.6% of patients had indications of a potential cognitive impairment by both PCI and TMT scores.

**TABLE 2:** Distribution of subjective and objective cognitive measures and percentage of patients with values indicating a potential cognitive impairment.

Measure [scale]	Median	Q1, Q3	Patients with values indicating a potential cognitive impairment	
			Proportion	Criterion/cut‐off
Subjective measures [0–100]				
FACT‐Cog, PCI	86.3	73.8, 95.0	25.1%	< 75 (i.e., 60 on raw scale, Van Dyk et al. 2020)
FACT‐Cog, PCA	83.3	63.9, 94.4	n.a.	
FACT‐Cog, IQoL	87.5	62.5, 100.0	n.a.	
EORTC QLQ‐C30, cognitive function	83.3	66.7, 100.0	38.4%	< 75 (Threshold of clinical importance, Giesinger et al. 2020)
Objective measures				
TMT‐A [s]	33.0	26.0, 43.0	8.9%	≥ 2 SD of normative mean^a^, ^c^
TMT‐B [s]	69.0	55.0, 90.0	10.6%	≥ 2 SD of normative mean^a^, ^c^
TMT overall			17.5%	At least one ≥ 2 SD or both ≥ 1.5 SD of normative mean^a^, ^c^
COWA phonemic fluency [words]	18	14, 23	n.a.	
COWA semantic fluency [words]	25	21, 29	n.a.	
HVLT‐R sum of learning trials [0–36]	30	20, 33	0.8%	≤ 2 SD of normative mean^b^, ^c^
HVLT‐R delayed recall [0–12]	11	9, 12	1.7%	≤ 2 SD of normative mean^b^, ^c^
HVLT‐R overall			4.3%	At least one ≥ 2 SD or both ≥ 1.5 SD of normative mean^b^, ^c^
HVLT‐R retention [%]	100.0	90.0, 100.0	n.a.	
HVLT‐R RDI [0–12]	10	11, 12	n.a.	

Abbreviations: COWA, Controlled Oral Word Association Test; FACT‐cog, Functional Assessment of Cancer Therapy‐cognitive scale; HVLT‐R, Hopkins Verbal Learning Test‐Revised; IQoL, Impact on quality of life; PCA, Perceived cognitive ability; PCI, Perceived cognitive impairment; Q1, Q3, 1st, 3rd percentile; RDI, Recognition Discrimination Index; SD, standard deviation; TMT, Trail Making Test.

^a^ Age‐ and education‐specific normative values of the TMT by Tombaugh et al. (2004).

^b^ Age‐specific normative values of COWA by Brandt und Benedict (2001).

^c^ Applying the criteria recommended by the International Cognition and Cancer Task Force for assessing cognitive impairment in cancer by Wefel et al. (2011).

The subjective measures correlated poorly with the objective measures. The partial Spearman correlation coefficients of the three subjective cognitive measures with the TMT and with HVLT‐R delayed recall and recognition discrimination index, controlling for age and education, were non‐significant or of low magnitude (all |*r*| ≤ 0.21, Table 3). There was no correlation of the subjective measures with COWA semantic or phonemic fluency. Partial Spearman correlations among subjective and among objective measures are presented in Table S2.

**TABLE 3:** Partial Spearman correlation between subjective and objective cognitive measures, adjusted for age and education.

Objective cognitive measures	Subjective cognitive measures: FACT‐Cog scores		
	Perceived cognitive impairment (PCI)	Perceived cognitive ability (PCA)	Impact on quality of life (IQoL)
TMT‐A	−0.15*	−0.14*	−0.15*
TMT‐B	−0.13	−0.18*	−0.21**
COWA, phonemic fluency test	0.06	0.07	0.06
COWA, semantic fluency test	0.07	0.10	0.03
HVLT‐R, sum of learning	0.07	0.07	0.08
HVLT‐R, delayed recall	0.18*	0.17*	0.20**
HVLT‐R, retention	0.12	0.12	0.12
HVLT‐R, RDI	0.12	0.14*	0.16*

*Note:* *p < 0.05, **p < 0.01.

Abbreviations: COWA, Controlled Oral Word Association Test; HVLT‐R, Hopkins Verbal Learning Test‐Revised; RDI, Recognition Discrimination Index; TMT, Trail Making Test.

### 3.3 Regression Analyses Investigating Potential Determinants of the Cognitive Measures

The results of the multiple linear regressions (Table 4, without depression and fatigue) showed that anxiety was a major factor associated with the three subscales of subjective CRCI: PCI [unstandardized estimate β = −0.33, 95% CI = (−0.44, −0.22)), PCA (−0.39 (−0.52, −0.26)] and IQoL [−0.78 (−0.94–0.61)]. Further, sleep problems and pain were significantly associated with PCI and PCA, and pain also with IQoL. Patients with basic education reported significantly lower PCA compared to patients with academic education (−8.98 (−16.2, −1.77)). Age, social support and other characteristics measured were not significantly associated with any of the 3 subscales of subjective CRCI. When exploring the models including also fatigue and depression in the models, only fatigue showed a significant association with PCI and PCA (Table S3). However, the causal direction between fatigue and CRCI is questionable.

**TABLE 4:** Multiple linear regression models investigating potential determinants of subjective cognitive function (FACT‐cog, *n* = 230).

	Perceived cognitive impairment (PCI)	Perceived cognitive ability (PCA)	Impact on quality of life (IQoL)
	β (95% CI)	β (95% CI)	β (95% CI)
Age	−0.02 (−0.17, 0.14)	0.05 (−0.13, 0.23)	0.15 (−0.09, 0.39)
Smoking			
Never	0.00 (Ref)	0.00 (Ref)	0.00 (Ref)
Former	1.50 (−2.18, 5.19)	2.41 (−1.83, 6.64)	1.18 (−4.43, 6.79)
Current	1.86 (−3.70, 7.41)	0.70 (−5.69, 7.09)	−0.00 (−8.46, 8.45)
Education			
Academic	0.00 (Ref)	0.00 (Ref)	0.00 (Ref)
High	2.91 (−1.75, 7.56)	1.20 (−4.15, 6.55)	1.82 (−5.32, 8.95)
Moderate	−2.53 (−6.71, 1.65)	−3.72 (−8.53, 1.09)	−3.95 (−10.3, 2.41)
Basic	−4.71 (−11.0, 1.56)	−8.98 (−16.2, −1.77)*	−4.80 (−14.3, 4.74)
Alcohol [g/day, log‐transformed]	−0.50 (−1.45, 0.45)	−0.08 (−1.18, 1.01)	−0.12 (−1.58, 1.34)
BMI [kg/m^2^]	−0.05 (−0.36, 0.25)	0.13 (−0.22, 0.48)	−0.08 (−0.55, 0.38)
Social support [0–100 scale]	0.08 (−0.06, 0.22)	0.07 (−0.09, 0.23)	0.01 (−0.21, 0.22)
Sleep problems [0–100 scale]	−0.16 (−0.28, −0.04)**	−0.14 (−0.28, −0.01)*	−0.03 (−0.21, 0.15)
Anxiety [0–100 scale]	−0.33 (−0.44, −0.22)***	−0.39 (−0.52, −0.26)***	−0.78 (−0.94, −0.61)***
Pain [0–100 scale]	−0.06 (−0.13, −0.00)*	−0.09 (−0.16, −0.02)*	−0.18 (−0.28, −0.09)***

*Note:* *p < 0.05, **p < 0.01, ***p < 0.001. Higher PCI, PCA, or IQoL scores indicate better cognitive function (i.e., lower subjective CRCI).

Abbreviations: β, unstandardized estimate; BMI, body mass index; CI, confidence interval; FACT‐cog, Functional Assessment of Cancer Therapy‐cognitive scale.

Regarding objective CRCI measures (Table 5), age appears to be a major influencing factor with higher age showing significant associations with poorer cognitive test results across all objective measures except for the phonemic fluency dimension of COWA. Moreover, academic education was associated with better cognitive test results than basic or moderate education for several of the objective measures. Poorer TMT‐B results were further significantly associated with higher levels of anxiety and pain and lower alcohol intake. Adding fatigue and depression to the model did not change the above noted results substantially (Table S4). Fatigue and depression showed no significant associations with the objective CRCI measures, even if only one of both factors was included.

**TABLE 5:** Multiple linear regression models investigating potential determinants of objectively measured cognitive function.

	Trail making test^a^ (TMT, *n* = 228)		Controlled oral word association test (COWA, *n* = 198)	
	TMT‐A	TMT‐B	Phonemic fluency	Semantic fluency
	β (95% CI)	β (95% CI)	β (95% CI)	β (95% CI)
Age	0.81 (0.62, 1.00)***	0.82 (0.64, 1.00)***	−0.03 (−0.12, 0.06)	−0.16 (−0.24, −0.09)***
Smoking				
Never	0.00 (Ref)	0.00 (Ref)	0.00 (Ref)	0.00 (Ref)
Former	−3.15 (−7.64, 1.34)	−0.43 (−4.76, 3.89)	0.26 (−1.97, 2.49)	1.11 (−0.66, 2.89)
Current	−0.76 (−7.51, 5.99)	3.32 (−3.31, 9.94)	−0.40 (−3.71, 2.91)	−1.01 (−3.65, 1.62)
Education				
Academic	0.00 (Ref)	0.00 (Ref)	0.00 (Ref)	0.00 (Ref)
High	−2.90 (−8.56, 2.76)	−0.67 (−6.11, 4.78)	−0.58 (−3.34, 2.18)	0.32 (−1.89, 2.53)
Moderate	−0.33 (−5.43, 4.78)	2.29 (−2.62, 7.20)	−4.73 (−7.19, −2.27)***	−0.90 (−2.86, 1.07)
Basic	−2.75 (−10.4, 4.85)	11.40 (3.94, 18.86)**	−1.81 (−5.55, 1.92)	−1.83 (−4.74, 1.09)
Alcohol [g/day, log‐transformed]	−0.70 (−1.85, 0.46)	−1.20 (−2.32, −0.09)*	0.26 (−0.31, 0.82)	0.02 (−0.43, 0.47)
BMI [kg/m^2^]	0.04 (−0.33, 0.41)	−0.10 (−0.47, 0.28)	−0.19 (−0.38, 0.00)	−0.04 (−0.19, 0.12)
Social support [0–100 scale]	−0.13 (−0.30, 0.05)	0.04 (−0.12, 0.21)	−0.02 (−0.10, 0.07)	0.08 (0.02, 0.15)*
Sleep problems [0–100 scale]	−0.01 (−0.15, 0.13)	−0.01 (−0.15, 0.12)	0.03 (−0.04, 0.11)	0.00 (−0.05, 0.06)
Anxiety [0–100 scale]	0.09 (−0.04, 0.22)	0.15 (0.02, 0.27)*	−0.02 (−0.08, 0.04)	0.01 (−0.04, 0.06)
Pain [0–100 scale]	0.08 (0.00, 0.15)*	0.10 (0.03, 0.17)**	0.00 (−0.04, 0.04)	0.01 (−0.02, 0.04)

	Hopkins verbal learning test‐revised (HVLT‐R, *n* = 228)			
	Sum of learning trials	Delayed recall	Retention	RDI
	β (95% CI)	β (95% CI)	β (95% CI)	β (95% CI)
Age	−0.07 (−0.12, −0.02)**	−0.04 (−0.06, −0.02)***	−0.20 (−0.35, −0.05)**	−0.02 (−0.03, −0.01)**
Smoking				
Never	0.00 (Ref)	0.00 (Ref)	0.00 (Ref)	0.00 (Ref)
Former	0.07 (−1.10, 1.24)	0.12 (−0.39, 0.63)	1.01 (−2.58, 4.60)	−0.08 (−0.37, 0.22)
Current	−0.30 (−2.06, 1.46)	0.05 (−0.72, 0.83)	0.68 (−4.77, 6.13)	−0.07 (−0.52, 0.37)
Education				
Academic	0.00 (Ref)	0.00 (Ref)	0.00 (Ref)	0.00 (Ref)
High	−0.05 (−1.53, 1.43)	−0.12 (−0.77, 0.53)	−2.01 (−6.56, 2.54)	−0.01 (−0.39, 0.36)
Moderate	−2.06 (−3.39, −0.73)**	−0.77 (−1.35, −0.19)**	−3.16 (−7.23, 0.92)	−0.31 (−0.64, 0.03)
Basic	−2.97 (−4.96, −0.98)**	−0.83 (−1.69, 0.04)	−2.70 (−8.83, 3.43)	−0.10 (−0.60, 0.41)
Alcohol [g/day, log‐transformed]	0.30 (−0.01, 0.60)	0.09 (−0.04, 0.22)	0.31 (−0.61, 1.24)	0.05 (−0.03, 0.13)
BMI [kg/m^2^]	−0.03 (−0.12, 0.07)	−0.00 (−0.04, 0.04)	0.14 (−0.16, 0.44)	0.00 (−0.02, 0.03)
Social support [0–100 scale]	−0.00 (−0.05, 0.04)	−0.01 (−0.03, 0.01)	−0.11 (−0.25, 0.04)	−0.00 (−0.01, 0.01)
Sleep problems [0–100 scale]	0.01 (−0.02, 0.05)	0.00 (−0.01, 0.02)	0.02 (−0.09, 0.13)	−0.00 (−0.01, 0.01)
Anxiety [0–100 scale]	−0.01 (−0.04, 0.03)	−0.01 (−0.03, 0.00)	−0.10 (−0.21, 0.00)	−0.01 (−0.02, 0.00)
Pain [0–100 scale]	−0.01 (−0.03, 0.01)	−0.00 (−0.01, 0.01)	−0.01 (−0.07, 0.05)	0.00 (−0.00, 0.01)

*Note:* *p < 0.05, **p < 0.01, ***p < 0.001.

Abbreviations: β, unstandardized estimate; BMI, body mass index; CI, confidence interval; RDI, Recognition Discrimination Index.

^a^ Log‐transformed.

Exploring further models where adding surgery (yes/no) as a covariate yielded a significant association of having had surgery with worse PCI and PCA without substantial changes in other estimates. However, only nine patients had no surgery. There were no significant associations of surgery with other subjective or any objective cognitive outcomes. Regarding the patients with surgery, time since surgery did not yield any significant association with subjective or objective cognitive outcomes.

## 4 Discussion

In this study, a quarter of the 239 included patients with breast or gynecological tumors showed indication for CRCI prior to the start of any systemic or radiation therapy, based on the subjective PCI measure. There were no or only weak correlations between subjective and objective measures of cognitive impairment. Subjective cognitive measures were strongly associated with anxiety and, to a lesser extent, with sleep problems and pain, whereas a major influencing factor of objective cognitive measures was age, followed by education.

Cognitive impairment encompasses a wide range of signs and problems, for example, reduced attention, impaired memory, word‐finding disorders, or “brain fog”, which all may have different pathophysiology. Likewise, there cannot be the ONE measure of cognitive impairment. To date, the exact meaning of the various measures and their relationship to each other is still under discussion. Hence, our findings, which align with prior research that has reported a weak correlation [8, 34, 35, 36] or even no association [37] between subjective and objective measures, are not irrational. Providing more insights into associated factors might help to put the measurements in the right context.

Our results strengthen the so‐far limited evidence that higher levels of anxiety were linked to worse subjective cognitive function in patients with breast or gynecological cancer [7, 38, 39, 40]. The relationship between cognitive impairment and anxiety may be bidirectional. Experiencing cognitive dysfunction may result in feelings of anxiety and depression. On the other hand, anxiety can promote a debilitating focus on negative life events, such as cancer, which can result in difficulties with concentration and other levels of cognition such as perception, memory, and executive function [41]. A study in 1332 cancer patients undergoing chemotherapy found that higher levels of CRCI co‐occurring with anxiety were associated with higher levels of stress and lower levels of resilience [40]. Moreover, gene expression analysis revealed that co‐occurring CRCI and anxiety were associated with perturbations in neurodegenerative disease pathways in patients receiving chemotherapy [40, 42]. In further studies, participants with (chronic) anxiety or worries experienced worse cognitive decline, faster brain aging, or impaired neurogenesis, showing poorer performance in memory tasks or brain structural differences, including reduced hippocampal volumes [42, 43, 44, 45]. Thus, providing psychological or mind–body interventions to reduce anxiety might mitigate the impact of perceived CRCI and potentially enhance overall well‐being [46].

Further, we found that having more sleep problems was associated with worse subjective cognitive functions. The findings corroborated other studies [7, 47, 48], which found that poor sleep quality and insomnia were associated with poor cognitive function in breast cancer patients. Also in non‐cancer patients as well as animal experiments, impaired sleep seemed to affect cognitive function via various mechanisms [49]. Previous experimental research in healthy volunteers likewise showed that sleep deprivation impairs working memory capacity, may lead to altered cerebral blood flow in the right middle cerebral artery, and may be associated with disturbed hemodynamic responses in the brain [50, 51]. Hence, good quality and sufficient sleep seem, therefore, essential for healthy neurocognitive function also in cancer patients. Therefore, sleep problems should be addressed and treated early in the cancer continuum.

Pain was another determinant of subjective CRCI in our as well as previous studies [52]. Associations of pain with subjective cognitive decline were also observed in non‐cancer situations [53]. It has been hypothesized that chronic pain might result in limited neural resources, neuroplastic changes, or dysregulated neurochemistry, which could impact cognitive functioning. Moreover, analgesics might also affect cognitive functioning.

Our data showed no association of social support with CRCI except for one of 11 measures, i.e., the semantic fluency test subdimension of COWA, suggesting that social support may have only a weak association with CRCI. While a study [39] with metastatic breast cancer patients discovered a correlation between higher social support and improved subjective CRCI, it did not find a similar correlation with objective CRCI.

Further, the observed anxiety‐sleep‐pain‐associated type of CRCI showed strong relations to fatigue. The causal relationship between CRCI and fatigue cannot be disentangled with our study. It likely is not unidirectional but complex. Indeed, cognitive exhaustion is one of the three dimensions of cancer‐related fatigue, and emotional distress (anxiety/depression), sleep problems, and pain often occur as a cluster together with fatigue [54]. As there were no or only weak associations of this perceived CRCI type with the objective measures, the ICCTF‐recommended neurocognitive tests seem not appropriate for capturing this type of CRCI.

Poor objective cognitive function measures were mainly associated with higher age, whereas there was no association of subjective CRCI measures with age. This age association of the objective measures is in line with previous studies [55, 56, 57], whereas one study [7] found that lower age was linked to worse subjective CRCI. Furthermore, in line with previous studies [57, 58], we found that higher education was associated with better objective CRCI. These age and education dependencies of the objective cognitive measures are also seen in normative values from the general population [21, 24]. In contrast, the subjectively assessed CRCI appeared to be largely independent of age or education.

### 4.1 Clinical Implications

Given the significant associations of perceived cognitive impairments before any systemic or radiation therapy with anxiety, sleep problems, and pain, it is important to assess and address these problems early during cancer treatment (ideally already before the initiation). Thus, psychosocial interventions targeting anxiety and sleep problems may play an important role in mitigating CRCI in cancer patients. Recent evidence highlights the effectiveness of mindfulness‐based stress reduction (MBSR) and brief acceptance and commitment therapy (ACT) in reducing CRCI, depression, and anxiety in breast cancer patients and survivors [46]. In case of sleep problems, cognitive behavioral therapy for insomnia (CBT‐I) is recommended by the ESMO Clinical Practice Guideline on insomnia in adult patients with cancer [59]. Exercise and yoga have also shown beneficial effects on anxiety, sleep problems, pain, as well as cognitive impairments, at least in specific subgroups [60, 61, 62]. In addition, potential negative impacts of analgesic drugs on cognition should be discussed with the patients to enable an informed decision.

Furthermore, considering factors such as age and education is crucial when interpreting the neuropsychological tests recommended by the ICCTF. Another study even suggested conducting a short IQ test for interpreting the neuropsychological test results [63]. Overall, these measures hence may not be appropriate for cross‐sectional assessments. They did not capture the above‐described pretreatment CRCI, emphasizing the need for healthcare providers to recognize that a patient's perception of cognitive impairment may not always align with objective assessments. It is important to consider that an individual's perception and report of cognitive impairments may reflect their real suffering. Lack of correlation between subjective and objective measures of CRCI may relate to an individual's cognitive reserve. Patients with high IQ or high education may have sufficient cognitive reserve to perform within normal limits on standard cognitive tests, while reporting significant subjective CRCI as they are expending substantial cognitive effort to accomplish the tasks. Moreover, it is possible that the neuropsychological cognitive tests, which are recommended by the ICCTF, may not be sensitive enough to capture cognitive problems of cancer patients, because they were originally developed to screen for or to measure more pronounced cognitive impairments than commonly experienced by cancer patients, such as dementia [10, 64]. On the other hand, these objective measures were less influenced by psychological factors. They could be useful to measure change over time or intervention effects, where with‐in person (pre‐/post‐intervention) changes in CRCI are considered. Hence, in such settings, the objective measures' dependence on individual characteristics (age, education, and others such as intelligence, verbal or visual capabilities) does not matter, and objective measures are less confounded by potential psychosocial effects. Therefore, both subjective and objective perspectives might be considered complementary in the evaluation and management of CRCI.

Moreover, a recent European Delphi study, which focused on enhancing the management of CRCI within clinical settings, delineated 15 expert‐endorsed suggestions [64]. Notably, the study advocates for a structured screening process utilizing subjective measures for CRCI, accompanied by a concise objective cognitive assessment in response to identified concerns. For persistent CRCI 6 months post‐treatment, the study advises a thorough evaluation, followed by the provision of cognitive rehabilitation, physical activity, meditative‐movement therapy, and multimodal interventions. Overall, these clinical implications stress the importance of a holistic and patient‐centred approach in addressing cognitive impairments in breast cancer patients. By considering these associated factors and prioritizing psychological interventions, healthcare providers can strive to enhance cognitive well‐being and overall quality of life for these patients.

### 4.2 Limitation, Strengths, and Suggestion for Future Studies

The study findings should be interpreted in light of certain limitations. The use of a cross‐sectional research design, which inherently lacks the ability to establish causality, is one such limitation. Additionally, our study was conducted within a single cancer center, potentially limiting the generalizability of the findings to the broader breast cancer population. As only nine patients had no cancer surgery, mainly because they were scheduled for neoadjuvant chemotherapy, this might have been a bit selective; we could not reliably investigate the effects of surgery on CRCI. Moreover, other determinants of CRCI may exist, e.g., post‐traumatic stress disorder symptoms, intelligence or cognitive reserve [1], which were not measured in the CogniFiT study. Despite these limitations, the study expands current knowledge. It goes beyond other studies in that it examined the same factors for both subjective and objective CRCI measures recommended by ICCTF in cancer patients before the start of systemic or radiation treatment. Our study indicates that a substantial number of patients experience CRCI that may be caused or related to anxiety/emotional stress, pain, sleep problems, or fatigue, and that this type of CRCI may not be well captured by the neuropsychological tests recommended by the ICCTF.

### 4.3 Conclusion

This study indicates that a substantial number of female cancer patients experience CRCI already prior to the initiation of any systemic or radiation therapy, which may be caused or related to anxiety/emotional stress, pain, sleep problems, or fatigue. Addressing these factors, ideally already prior to or early in cancer therapy, might help to reduce cognitive problems. The neuropsychological tests recommended by the ICCTF might not reliably capture this form of CRCI. However, the objective cognitive measures might be used in a complementary way to assess changes in cognitive function over time, unaffected by psychosocial influences.

## Author Contributions

**Maria‐Chidi C. Onyedibe:** writing – original draft, writing – review and editing, formal analysis. **Martina E. Schmidt:** conceptualization, data curation, investigation, formal analysis, writing – original draft, writing – review and editing, supervision. **Pauline Bizer:** data curation, investigation, writing – review and editing. **Philipp Zimmer:** conceptualization, writing – review and editing, project administration, investigation. **Karen Steindorf:** conceptualization, writing – review and editing, project administration, supervision, investigation.

## Ethics Statement

This study was conducted in accordance with the ethical standards of the Helsinki Declaration. The study was approved by the Ethic Committee of the Medical Faculty of the University of Heidelberg. All patients have given written informed consent. The study was registered at the German clinical trial registry (DRKS00015757, Deutsches Register Klinischer Studien).

## Conflicts of Interest

The authors declare no conflicts of interest.

## Supporting information


**Table S1** Spearman correlation of subjective and objective cognitive measures with depressive symptoms and fatigue.

**Table S2** Partial Spearman correlation between all cognitive measures, controlling for age and education.

**Table S3** Multiple linear regression models investigating potential determinants of subjective cognitive function, including fatigue, and depression (FACT‐cog, *n* = 230).

**Table S4** Multiple linear regression models investigating potential determinants of objectively measured cognitive function, including fatigue and depression.

## Data Availability

The data that support the findings of this study are available on request from the corresponding author. The data are not publicly available due to privacy or ethical restrictions.
