# Supplementary material for: Subjective and Objective Cancer‐Related Cognitive Impairments Among Systemic and Radiation Therapy‐Naïve Female Cancer Patients
Source: Cancer Med. 2025 Apr 22;14(8):e70908. doi: 10.1002/cam4.70908 (PMC12012307; doi:10.1002/cam4.70908)
Supplement: Supplementary file 1 — Table S1 Spearman correlation of subjective and objective cognitive measures with depressive symptoms and fatigue. [file CAM4-14-e70908-s004.docx]

**Supplemental Table S1:** Spearman correlation of subjective and objective cognitive measures with depressive symptoms and fatigue

|  |  | **Depression CES-D total score** | **Fatigue**  **EORTC QLQ-FA12 total** |
| --- | --- | --- | --- |
| **FACT-cog** | **PCI: Perceived cognitive impairment** | -0.63*** | -0.72*** |
|  | **PCA: Perceived cognitive ability** | -0.62*** | -0.71*** |
|  | **IQoL: Impact on quality of life** | -0.66*** | -0.66*** |
| **TMT** | **A** | 0.12 | 0.15* |
|  | **B** | 0.24** | 0.26*** |
| **HVLT-R** | **Sum of learning** | -0.07 | -0.11 |
|  | **Delayed recall** | -0.17* | -0.19* |
|  | **Retention** | -0.09 | -0.12 |
|  | **RDI: Recognition Discrimination Index** | -0.19* | -0.19* |
| **COWA** | **Phonemic fluency** | 0.01 | -0.02 |
|  | **Semantic fluency** | 0.00 | -0.05 |

* P < .05, ** p<.001, *** P < .0001

COWA: Controlled Oral Word Association Test; FACT-cog: Functional Assessment of Cancer Therapy – cognitive scale; HVLT-R: Hopkins Verbal Learning Test-Revised; TMT: Trail Making Test
