# Supplementary material for: Subjective and Objective Cancer‐Related Cognitive Impairments Among Systemic and Radiation Therapy‐Naïve Female Cancer Patients
Source: Cancer Med. 2025 Apr 22;14(8):e70908. doi: 10.1002/cam4.70908 (PMC12012307; doi:10.1002/cam4.70908)
Supplement: Supplementary file 2 — Table S2 Partial Spearman correlation between all cognitive measures, controlling for age and education. [file CAM4-14-e70908-s001.docx]

**Supplemental Table S2:** Partial Spearman correlation between all cognitive measures, controlling for age and education

|  |  | **TMT** | | **HVLT-R** | | | | **COWA** | | **FACT-cog** | | |
| --- | --- | --- | --- | --- | --- | --- | --- | --- | --- | --- | --- | --- |
|  |  | **A** | **B** | **Sum of learning** | **Delayed recall** | **Retention** | **RDI** | **Phonemic fluency** | **Semantic fluency** | **PCI** | **PCA** | **IQoL** |
| **TMT** | **A** | 1.00 | 0.50 | -0.13 | -0.16 | -0.11 | -0.15 | -0.28 | -0.18 | -0.15 | -0.14 | -0.15 |
|  | **B** | 0.50 | 1.00 | -0.24 | -0.16 | -0.06 | -0.18 | -0.20 | -0.31 | -0.13 | -0.18 | -0.21 |
| **HVLT-R** | **Sum of learning** | -0.13 | -0.24 | 1.00 | 0.70 | 0.23 | 0.41 | 0.09 | 0.27 | 0.07 | 0.07 | 0.08 |
|  | **Delayed recall** | -0.16 | -0.16 | 0.70 | 1.00 | 0.71 | 0.48 | 0.09 | 0.22 | 0.18 | 0.17 | 0.20 |
|  | **Retention** | -0.11 | -0.06 | 0.23 | 0.71 | 1.00 | 0.30 | 0.05 | 0.14 | 0.12 | 0.12 | 0.12 |
|  | **RDI** | -0.15 | -0.18 | 0.41 | 0.48 | 0.30 | 1.00 | 0.04 | 0.04 | 0.12 | 0.14 | 0.16 |
| **COWA** | **Phonemic fluency** | -0.28 | -0.20 | 0.09 | 0.09 | 0.05 | 0.04 | 1.00 | 0.35 | 0.06 | 0.07 | 0.06 |
|  | **Semantic fluency** | -0.18 | -0.31 | 0.27 | 0.22 | 0.14 | 0.04 | 0.35 | 1.00 | 0.07 | 0.10 | 0.03 |
| **FACT-cog** | **PCI** | -0.15 | -0.13 | 0.07 | 0.18 | 0.12 | 0.12 | 0.06 | 0.07 | 1.00 | 0.85 | 0.58 |
|  | **PCA** | -0.14 | -0.18 | 0.07 | 0.17 | 0.12 | 0.14 | 0.07 | 0.10 | 0.85 | 1.00 | 0.58 |
|  | **IQoL** | -0.15 | -0.21 | 0.08 | 0.20 | 0.12 | 0.16 | 0.06 | 0.03 | 0.58 | 0.58 | 1.00 |

COWA: Controlled Oral Word Association Test; FACT-cog: Functional Assessment of Cancer Therapy – cognitive scale; HVLT-R: Hopkins Verbal Learning Test-Revised; IQoL: Impact on quality of life; PCA: Perceived cognitive ability; PCI: Perceived cognitive impairment; RDI: Recognition Discrimination Index; TMT: Trail Making Test
