# Supplementary material for: Subjective and Objective Cancer‐Related Cognitive Impairments Among Systemic and Radiation Therapy‐Naïve Female Cancer Patients
Source: Cancer Med. 2025 Apr 22;14(8):e70908. doi: 10.1002/cam4.70908 (PMC12012307; doi:10.1002/cam4.70908)
Supplement: Supplementary file 3 — Table S3 Multiple linear regression models investigating potential determinants of subjective cognitive function, including fatigue, and depression (FACT‐cog, n = 230). [file CAM4-14-e70908-s003.docx]

**Table S3:** Multiple linear regression models investigating potential determinants of subjective cognitive function, including fatigue and depression (FACT-cog, n = 230)

|  | **Perceived cognitive impairment (PCI)** | **Perceived cognitive ability (PCA)** | **Impact on quality of life (IQoL)** |
| --- | --- | --- | --- |
|  | **β (95% CI)** | **β (95% CI)** | **β (95% CI)** |
| **Age** | -0.01 (-0.15, 0.13) | 0.06 (-0.09, 0.22) | 0.14 (-0.08, 0.36) |
| **Smoking:** never | 0.00 (Ref) | 0.00 (Ref) | 0.00 (Ref) |
| former | 0.74 (-2.65, 4.13) | 1.45 (-2.28, 5.18) | 1.45 (-3.87, 6.77) |
| current | 3.04 (-1.67, 7.75) | 2.68 (-2.49, 7.86) | 0.21 (-7.16, 7.57) |
| **Education:** academic | 0.00 (Ref) | 0.00 (Ref) | 0.00 (Ref) |
| high | 3.10 (-1.12, 7.32) | 1.52 (-3.12, 6.15) | 1.59 (-5.06, 8.23) |
| moderate | -2.11 (-5.90, 1.69) | -3.16 (-7.33, 1.01) | -3.46 (-9.39, 2.48) |
| basic | -3.04 (-8.73, 2.65) | -7.10 (-13.4, -0.84)* | -1.30 (-10.2, 7.60) |
| **Alcohol** [g/day, log-transformed] | -0.45 (-1.31, 0.42) | -0.01 (-0.96, 0.94) | -0.20 (-1.56, 1.17) |
| **BMI** [kg/m^2^] | 0.07 (-0.21, 0.34) | 0.29 (-0.02, 0.59) | 0.09 (-0.34, 0.53) |
| **Social support** [0-100 scale] | 0.05 (-0.08, 0.18) | 0.03 (-0.11, 0.18) | -0.01 (-0.21, 0.20) |
| **Sleep problems** [0-100 scale] | -0.08 (-0.19, 0.04) | -0.04 (-0.17, 0.08) | 0.13 (-0.05, 0.31) |
| **Anxiety** [0-100 scale] | 0.02 (-0.13, 0.16) | 0.06 (-0.10, 0.22) | -0.27 (-0.49, -0.04)* |
| **Pain** [0-100 scale] | 0.01 (-0.05, 0.07) | 0.02 (-0.05, 0.08) | -0.08 (-0.18, 0.01) |
| **Fatigue** [0-100 scale] | -0.43 (-0.57, -0.28)*** | -0.60 (-0.76, -0.44)*** | -0.38 (-0.61, -0.15)** |
| **Depression** [0-100 scale] | -0.05 (-0.23, 0.13) | -0.01 (-0.21, 0.19) | -0.38 (-0.66, -0.10)** |

٭ p<.05, ٭٭ p<.01, ٭٭٭ p<.001, BMI= body mass index; β: unstandardized estimate; CI: confidence interval; FACT-cog: Functional Assessment of Cancer Therapy – cognitive scale
Note: Higher PCI, PCA, or IQoL scores indicating better cognitive function (i.e., lower subjective CRCI). Fatigue assessed by EORTC QLQ-FA12, total fatigue score; Depression assessed by CES-D.
