# Supplementary material for: Subjective and Objective Cancer‐Related Cognitive Impairments Among Systemic and Radiation Therapy‐Naïve Female Cancer Patients
Source: Cancer Med. 2025 Apr 22;14(8):e70908. doi: 10.1002/cam4.70908 (PMC12012307; doi:10.1002/cam4.70908)
Supplement: Supplementary file 4 — Table S4 Multiple linear regression models investigating potential determinants of objectively measured cognitive function, including fatigue and depression. [file CAM4-14-e70908-s002.docx]

**Table S4:** Multiple linear regression models investigating potential determinants of objectively measured cognitive function**, including fatigue and depression**

|  | **Trail Making Test^a^** (TMT, n=228) | | **Controlled Oral Word Association Test** (COWA, n=198) | |
| --- | --- | --- | --- | --- |
|  | **TMT-A** | **TMT-B** | **Phonemic fluency** | **Semantic fluency** |
|  | **β (95% CI)** | **β (95% CI)** | **β (95% CI)** | **β (95% CI)** |
| **Age** | 0.79 (0.60, 0.98)*** | 0.81 (0.63, 0.99)*** | -0.03 (-0.12, 0.06) | -0.16 (-0.23, -0.09)*** |
| **Smoking:** never | 0.00 (Ref) | 0.00 (Ref) | 0.00 (Ref) | 0.00 (Ref) |
| former | -2.80 (-7.35, 1.75) | -0.20 (-4.60, 4.20) | 0.16 (-2.11, 2.43) | 0.83 (-0.99, 2.64) |
| current | -2.84 (-9.15, 3.47) | 1.37 (-4.82, 7.55) | -0.40 (-3.48, 2.67) | -0.56 (-3.02, 1.91) |
| **Education:** academic | 0.00 (Ref) | 0.00 (Ref) | 0.00 (Ref) | 0.00 (Ref) |
| high | -2.94 (-8.60, 2.73) | -0.56 (-6.03, 4.91) | -0.51 (-3.27, 2.25) | 0.37 (-1.85, 2.59) |
| moderate | -0.25 (-5.36, 4.87) | 2.14 (-2.79, 7.07) | -4.72 (-7.18, -2.25)*** | -0.89 (-2.87, 1.09) |
| basic | -1.18 (-8.80, 6.44) | 11.15 (3.65, 18.66)** | -2.35 (-6.13, 1.43) | -2.21 (-5.17, 0.75) |
| **Alcohol** [g/day, log-transformed] | -0.73 (-1.89, 0.43) | -1.23 (-2.35, -0.11)* | 0.28 (-0.29, 0.85) | 0.01 (-0.45, 0.46) |
| **BMI** [kg/m^2^] | 0.07 (-0.30, 0.44) | -0.15 (-0.52, 0.23) | -0.20 (-0.39, -0.01)* | -0.03 (-0.19, 0.12) |
| **Social support** [0-100 scale] | -0.11 (-0.29, 0.06) | 0.05 (-0.12, 0.21) | -0.02 (-0.11, 0.06) | 0.08 (0.01, 0.14)* |
| **Sleep problems** [0-100 scale] | 0.04 (-0.11, 0.19) | -0.05 (-0.19, 0.10) | 0.01 (-0.07, 0.09) | -0.01 (-0.07, 0.05) |
| **Anxiety** [0-100 scale] | 0.19 (-0.00, 0.39) | 0.03 (-0.16, 0.22) | -0.06 (-0.15, 0.04) | -0.01 (-0.08, 0.07) |
| **Pain** [0-100 scale] | 0.09 (0.01, 0.17)* | 0.08 (-0.00, 0.15) | -0.00 (-0.04, 0.04) | 0.01 (-0.02, 0.04) |
| **Fatigue** [0-100 scale] | 0.04 (-0.16, 0.24) | 0.11 (-0.08, 0.30) | -0.03 (-0.12, 0.07) | -0.03 (-0.11, 0.04) |
| **Depression** [0-100 scale] | -0.21 (-0.46, 0.03) | 0.06 (-0.18, 0.29) | 0.09 (-0.03, 0.21) | 0.06 (-0.03, 0.16) |
|  | **Hopkins Verbal Learning Test-Revised** (HVLT-R, n=228) | | | |
| (continued) | **Sum of learning trials** | **Delayed recall** | **Retention** | **RDI** |
|  | **β (95% CI)** | **β (95% CI)** | **β (95% CI)** | **β (95% CI)** |
| **Age** | -0.06 (-0.11, -0.01)* | -0.04 (-0.06, -0.02)*** | -0.20 (-0.35, -0.04)* | -0.02 (-0.03, -0.01)** |
| **Smoking:** never | 0.00 (Ref) | 0.00 (Ref) | 0.00 (Ref) | 0.00 (Ref) |
| former | -0.11 (-1.30, 1.08) | 0.07 (-0.45, 0.59) | 0.85 (-2.82, 4.52) | -0.10 (-0.40, 0.20) |
| current | 0.38 (-1.27, 2.03) | 0.32 (-0.40, 1.04) | 1.77 (-3.35, 6.89) | 0.06 (-0.36, 0.48) |
| **Education:** academic | 0.00 (Ref) | 0.00 (Ref) | 0.00 (Ref) | 0.00 (Ref) |
| high | -0.13 (-1.61, 1.36) | -0.15 (-0.80, 0.50) | -2.05 (-6.63, 2.54) | -0.04 (-0.41, 0.34) |
| moderate | -2.04 (-3.38, -0.70)** | -0.77 (-1.35, -0.19)** | -3.15 (-7.25, 0.96) | -0.31 (-0.65, 0.03) |
| basic | -2.92 (-4.91, -0.92)** | -0.81 (-1.68, 0.06) | -2.94 (-9.12, 3.24) | -0.08 (-0.59, 0.43) |
| **Alcohol** [g/day, log-transformed] | 0.29 (-0.01, 0.60) | 0.09 (-0.04, 0.23) | 0.33 (-0.60, 1.27) | 0.05 (-0.03, 0.13) |
| **BMI** [kg/m^2^] | -0.02 (-0.12, 0.08) | 0.00 (-0.04, 0.04) | 0.14 (-0.16, 0.44) | 0.00 (-0.02, 0.03) |
| **Social support** [0-100 scale] | -0.00 (-0.05, 0.04) | -0.01 (-0.03, 0.01) | -0.11 (-0.25, 0.03) | -0.00 (-0.01, 0.01) |
| **Sleep problems** [0-100 scale] | 0.02 (-0.02, 0.06) | 0.01 (-0.01, 0.02) | 0.02 (-0.10, 0.14) | -0.00 (-0.01, 0.01) |
| **Anxiety** [0-100 scale] | 0.02 (-0.03, 0.07) | -0.00 (-0.03, 0.02) | -0.09 (-0.25, 0.07) | -0.00 (-0.02, 0.01) |
| **Pain** [0-100 scale] | -0.00 (-0.02, 0.02) | 0.00 (-0.01, 0.01) | -0.01 (-0.07, 0.06) | 0.00 (-0.00, 0.01) |

٭ p<.05, ٭٭ p<.01, ٭٭٭ p<.001, BMI= body mass index; β: unstandardized estimate; CI: confidence interval, RDI: Recognition Discrimination Index

^a^ log-transformed
Note: Fatigue assessed by EORTC QLQ-FA12, total fatigue score; Depression assessed by CESD-R.
